# Signaling Pathways mTOR and ERK as Therapeutic Targets in Sinonasal Intestinal-Type Adenocarcinoma

**DOI:** 10.3390/ijms242015110

**Published:** 2023-10-12

**Authors:** Helena Codina-Martínez, Sara Lucila Lorenzo-Guerra, Virginia N. Cabal, Rocío García-Marín, Laura Suárez-Fernández, Blanca Vivanco, Paula Sánchez-Fernández, Fernando López, José Luis Llorente, Mario A. Hermsen

**Affiliations:** 1 Department of Head and Neck Cancer, Instituto de Investigación Sanitaria del Principado de Asturias, 33011 Oviedo, Spain; helenacm14@gmail.com (H.C.-M.); saralgcv96@gmail.com (S.L.L.-G.); vircabal@hotmail.com (V.N.C.); rociogm220879@hotmail.com (R.G.-M.); suarezflaura@gmail.com (L.S.-F.); 2 Department of Pathology, Hospital Universitario Central de Asturias, 33011 Oviedo, Spain; vivancoblanca@gmail.com; 3 Department of Otolaryngology, Hospital Universitario Central de Asturias, 33011 Oviedo, Spain; psfernan90@hotmail.com (P.S.-F.); flopez_1981@yahoo.es (F.L.); llorentejlx@gmail.com (J.L.L.)

**Keywords:** sinonasal cancer, intestinal-type adenocarcinoma, mTOR, ERK, preclinical model, personalized therapy

## Abstract

Despite advances in surgery and radiotherapy, the overall prognosis of sinonasal intestinal-type adenocarcinoma (ITAC) is poor, and new treatment options are needed. Recent studies have indicated alterations in cellular signaling pathways that may serve as targets for modern inhibitors. Our aim was to evaluate the frequency of mTOR and ERK pathway upregulation in a retrospective series of 139 ITAC and to test the efficacy and mechanism of action of candidate targeted inhibitors in cell line ITAC-3. An immunohistochemical analysis on p-AKT, p-mTOR, p-S6, p-4E-BP1, and p-ERK indicated, respectively, a 68% and 57% mTOR and ERK pathway activation. In vitro studies using low doses of mTOR inhibitor everolimus and ERK inhibitor selumetinib showed significant growth inhibition as monotherapy and especially as combined therapy. This effect was accompanied by the downregulation of mTOR and ERK protein expression. Our data open a new and promising possibility for personalized treatment of ITAC patients.

## 1. Introduction

Intestinal-type sinonasal adenocarcinoma (ITAC) is a rare tumor predominantly occurring in men with an occupational exposure to wood and leather dust [1,2,3,4,5]. The incidence is variable between different countries, being significantly higher in Europe with 0.3/100,000 inhabitants compared to North America with 0.092/100,000 inhabitants [1,5,6,7,8]. Unfortunately, there is a lack of published reports on ITAC in other parts of the world. ITAC is histologically similar to colorectal adenocarcinoma [2,6,9], but it arises in the respiratory and olfactory mucosa in the ethmoid sinus through a process of transdifferentiation [10,11]. As in colorectal adenocarcinoma, different histological subtypes of ITAC exist, with solid and mucinous types having a worse clinical course than papillary and colonic types [6,7]. The standard of treatment of ITAC is surgery followed by radiotherapy and sometimes chemotherapy [1,3,12,13]. Despite advances in these approaches, such as transnasal endoscopic surgery, intensity-modulated radiation therapy, volumetric modulated arc therapy, and heavy-particle therapy [14,15,16,17], the overall prognosis is poor. Five-year overall survival ranges from 80% in stage I tumors to 30% in stage IV tumors, and the main cause of mortality is local recurrence, occurring in 40–50% of all cases [1,3,18,19]. Therefore, there is a great need for new treatment options. Recent studies have indicated a number of genetic alterations in cellular signaling pathways that may serve as targets for modern inhibitors [20,21,22,23,24,25,26].

**Figure 1:**
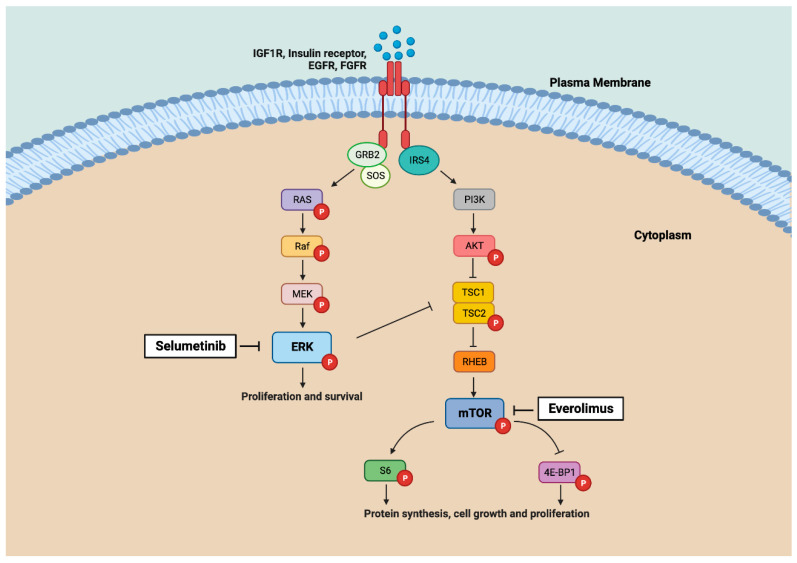
Map of mTOR and ERK signalling pathways showing genes and proteins investigated in this study. Selumetinib and everolimus inhibitors inactivate by acting on targets ERK and mTOR, respectively. Figure created with BioRender.com.

One of the most studied therapeutic targets in cancer is mTOR signaling, which plays a role in various cellular processes, including proliferation, differentiation, metabolism, genomic instability, and angiogenesis [27]. Genetic alterations in this molecular pathway occur in head and neck cancers [28,29] and have recently also been shown in ITAC [20,21,22]. In addition, an immunohistochemical analysis revealed increased levels of mTOR activation and downstream effectors EIF2S1 and EIF6 in a majority of cases [21,22]. In addition to mTOR, the ERK (or MAPK) pathway is also involved in cell proliferation and survival (Figure 1), and the inhibition of mTOR may cause induction of ERK leading to drug resistance [30,31]. For this reason, both pathways must be studied together. Previous studies have also shown ERK pathway gene mutations and overexpression in a substantial percentage of ITAC [20,21].

We hypothesized that the mTOR and ERK pathways could be used as therapeutic targets for the treatment of a subset of ITAC patients. Here, we evaluated a large series of 139 ITAC and confirmed that both mTOR and ERK pathways are frequently activated. In addition, we used tumor cell line ITAC-3 to study the growth inhibition effects of mTOR and ERK inhibitors in vitro. To our knowledge, this is the first study focusing on these pathways to test novel therapeutic options for ITAC patients.

## 2. Results

### 2.1. mTOR and ERK Pathways Are Frequently Activated in ITAC

**Figure 2:**
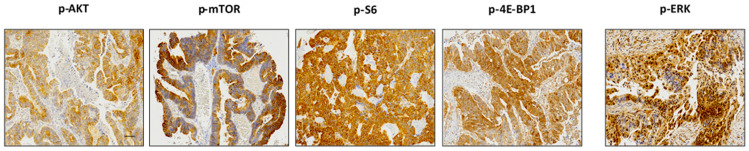
Representative immunohistochemical staining of p-AKT, p-mTOR, p-S6, p-4E-BP1, and p-ERK. Magnification 20×.

The expression of several proteins involved in mTOR signalling was evaluated by immunohistochemistry. p-AKT staining was observed in 70% (47/67) of the cases, of which 48% had low, 25% had medium, and 27% had high scores. p-mTOR expression occurred in 90% (125/139) of cases, with 32% with low, 32% with medium, and 36% with high positivity. All except 1 of 118 cases were positive for p-S6: 29% scored low, 28% scored medium, and 47% scored high. In addition, p-4E-BP1 was found expressed in a majority of cases (111/118, 94%), with 26% of cases presenting low, 35% presenting medium, and 39% presenting high scores. The activity of ERK pathway signalling was evaluated with p-ERK only, which showed positivity in 82% (113/138) of cases: 44% scored low, 28% scored medium, and 28% scored high. Representative examples of each staining are given in Figure 2.

Expression scores of p-mTOR, p-S6, p-4E-BP1, and p-ERK were interrelated so that cases with a high score for one of these proteins also had a high expression of the others. In contrast, the expression levels of p-AKT did not correlate with those of the other proteins.

All results were studied for possible correlation to clinico-pathological characteristics, including tumor stage, histological subtype, recurrence, metastasis, and patient status (Table 1). ITAC colonic and solid subtypes showed significantly more p-AKT expression that papillary and mucinous tumors (Pearson Chi2 *p* = 0.018). High expression scores of all evaluated proteins correlated with an absence of metastasis during follow-up. However, none of the staining was associated with tumor stage, recurrence, or patient status.

### 2.2. Both mTOR and ERK Pathways Are Activated in Cell Line ITAC-3

**Figure 3:**
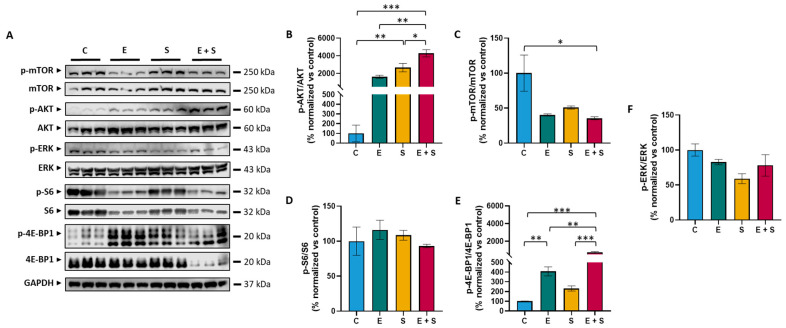
Representative immunoblots for p-AKT, AKT, p-mTOR, mTOR, p-S6, S6, p-4E-BP1, 4E-BP1, p-ERK, and ERK expression in cell line ITAC-3 after 24 h with everolimus and selumetinib treatment (**A**). Expression ratios of phosphorylated and unphosphorylated AKT (**B**), mTOR (**C**), S6 (**D**), 4E-BP1 (**E**), and ERK (**F**) upon exposure to everolimus, selumetinib, and a combination of everolimus and selumetinib (all at 50 nM dosis), normalized to unexposed control cells. Experiments carried out in triplicate; expression ratios are shown as mean values ± standard error of the mean. Legend. C: unexposed control cells; E: everolimus at 50 nM dosis; S: selumetinib at 50 nM dosis; E + S: everolimus and selumetinib both at 50 nM dosis; * *p* = 0.05–0.01; ** *p* = 0.01–0.001; *** *p* < 0.001.

A WES analysis of cell line ITAC-3 revealed two alterations in genes involved in the mTOR pathway; one concerned a frameshift mutation affecting exon 1 of *IRS4* (c.2244delC) (Supplementary Figure S1) and the other *TSC1*, which suffered a heterozygous deletion. The Western blot analysis showed strong expression levels of p-mTOR, p-S6, and p-ERK; low expression of p-4E-BP1; and an almost complete absence of p-AKT (Figure 3). These results indicate that cell line ITAC-3 is useful as a preclinical model to test mTOR and ERK pathway inhibitors.

### 2.3. Everolimus and Selumetinib Inhibit mTOR and ERK Signalling and Cell Proliferation of Cell Line ITAC-3

**Figure 4:**
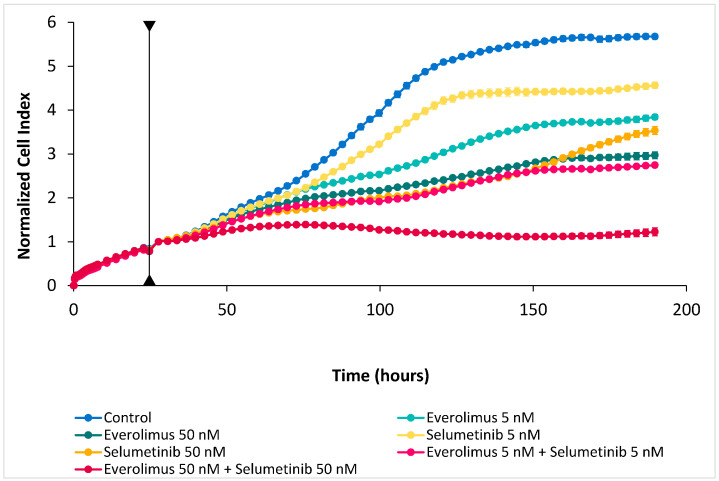
Cell proliferation of ITAC-3 measured in real time. Data are normalized from the time at which the inhibitor was added (vertical line). Experiments carried out in triplicate, mean values ± standard deviation.

We tested the growth inhibitory potential of mTOR inhibitor everolimus and ERK inhibitor selumetinib as monotherapy and as a combination at concentrations of 5 nM and 50 nM using real-time monitoring up to 190 h. The results showed reduced cell proliferation for all exposure schemes (Figure 4). Everolimus produced a stronger inhibition than selumetinib, and the effect was dose-related for both drugs. A combination at 5 nM doses showed a stronger growth reduction than the mono-exposure of both inhibitors at 50 nM doses while a combinatory exposure to 50 nM doses almost completely stopped cell proliferation.

Based on these results, we chose 24 and 48 h exposures to everolimus and selumetinib at 50 nM doses to analyze their effect on protein levels of mTOR and ERK pathways by Western blot (Figure 3 and Figure S2). We found p-mTOR/mTOR ratios significantly decreased by 24 h with combined exposure to everolimus and selumetinib, and a similar trend was observed by treatment with either everolimus or selumetinib. However, after 48 h of exposure, p-mTOR/mTOR levels were comparable with unexposed control cells. The downstream factor p-4E-BP1 showed a clear increase in expression both at 24 and 48 h exposure to everolimus and a very strong increase to combined exposure. The expression of the other downstream factor, p-S6, did not change in response to any of the treatment schemes. Upstream factor p-AKT/AKT ratios showed significant increases upon 24 h exposure to everolimus, selumetinib, and their combination while at 48 h, this effect was mostly lost. Finally, none of the treatment schemes produced a decrease in the p-ERK/ERK expression ratio at 24 h, but at 48 h, there were modest decreases, particularly with mono-exposure to selumetinib.

## 3. Discussion

The mTOR and ERK pathways are potential therapeutic targets for many types of cancer. Gene mutations have been reported, and signalling is frequently deregulated and hyperactivated, including in head and neck cancers and ITAC [20,21,22,27,28,29]. The ERK pathway, another frequently altered pathway in cancer, has common inputs with mTOR and has been shown to produce compensatory effects upon mTOR inhibition [30,31].

In the present study, we demonstrated a high incidence of both mTOR and ERK pathway activation in a large series of ITAC. The distribution of stage and histological subtypes as well as the rate of recurrence and metastasis is comparable to other published series [1,3,7,15,19]. In addition to metabolism and cell cycle regulation, both pathways have been suggested to also play a role in metastasis [27,32]. Our immunohistochemical data, however, showed that all four phosporylated mTOR pathway proteins as well as p-ERK correlate with the absence of distant metastasis (Table 1), so this finding will need additional studies. Irrespective of the role of mTOR and ERK signalling in metastasis, our data indicate that both are promising targets for new therapeutic options for ITAC patients.

Aiming to test such therapies in a preclinical setting, we used tumor cell line ITAC-3. This cell line was established and published by our group in 2011 and, unfortunately, still remains the only available preclinical model of ITAC to date [33]. A WES analysis did not reveal a mutation in key players of mTOR or ERK pathways; however, we did find a mutation in *IRS4*. This gene is part of the *IRS* family (*IRS1* to *IRS6*), which are adaptor proteins involved in insulin receptors and IGF1R. Although one of the least characterized proteins in this family, IRS4 has been associated with the hyperactivation of the mTOR and ERK pathways in lung and breast cancer [34,35]. We also found a heterozygous deletion of *TSC1* in ITAC-3 cells. Previous studies on ITAC have reported *TSC2* mutations [20,21], which suggest that ITAC alterations in the TSC complex may be involved in the upregulation of mTOR (Figure 1). The suitability of cell line ITAC-3 to test mTOR and ERK pathway inhibitors was confirmed with a Western blot analysis of untreated cells showing high expression levels of activated (i.e., phosphorylated) mTOR, S6, and ERK proteins and a low expression of AKT and 4E-BP1 (Figure 3).

We chose to test the efficacy to reduce cell proliferation of everolimus and selumetinib as inhibitors of mTOR and ERK pathways, respectively. These drugs suppress two different but interconnected signalling pathways. Everolimus acts directly on mTOR and is approved for lung, gastrointestinal, neuroendocrine tumors, and advanced renal carcinoma while selumetinib directly inhibits ERK, and its application is approved for neurofibromas [36,37]. In addition, both compounds are being tested in various clinical trials on non-small cell lung, thyroid, and colorectal cancer, among others [38]. Our results on ITAC-3 cells showed a clear inhibitory effect with everolimus, even as a monotherapy at lower concentrations than described in similar studies on colon and breast cancer cell lines [39,40]. Exposure to selumetinib also caused an inhibition of cell growth as has been shown in cell lines of other cancer types [41,42]. However, a combination of both inhibitors showed the strongest growth reduction and was clearly superior to monotherapy, as has been shown for other cancer types [31].

The Western blot expression analysis of mTOR pathway proteins demonstrated the expected decrease in p-mTOR and increase in p-4E-BP1 at 24 h exposure to everolimus. These effects were mostly lost at 48 h; therefore, we speculate that the observed p-AKT upregulation may be responsible for the reduced mTOR signalling seen at 48 h and could indicate a mechanism of resistance to therapy [43]. The Western blot data also confirmed that exposure to selumetinib causes the expected decrease in p-ERK ratio levels and, similar to everolimus treatment, leads to an upregulation of p-AKT. Surprisingly, everolimus treatment also resulted in decreased ERK levels at 48 h while 24 h of exposure selumetinib led to reduced p-mTOR expression. This underlines the complex interconnection between both pathways. Compared to these monotherapy analyses, the strongest downregulation of p-mTOR and upregulation of p-4E-BP1 and p-AKT was observed with combined exposure to everolimus and selumetinib, which concurred with the strongest inhibition of cell proliferation.

In conclusion, there is an unmet clinical need for alternative therapies for ITAC patients, particularly for those with recurrent and metastatic diseases. We found that mTOR and ERK pathways are activated in a large proportion of cases and may serve as targets for personalized therapy. Using cell line ITAC-3 as a preclinical model for growth inhibition studies, we showed that inhibitors of these two pathways indeed have a cytostatic effect, which is accompanied by the downregulation of mTOR and ERK signalling, particularly with the combination treatment. Our data may open a new and promising possibility for personalized treatment of ITAC patients.

## 4. Materials and Methods

### 4.1. Primary Tumor Samples and Cell Line

Tissue microarrays (TMAs) were created of 139 primary ITAC samples and 6 normal mucosa controls [23]. The mean age was 65 years (range 28–92), 136 were male, and 3 were female. An amount of 31 cases were stage I, 17 stage II, 45 stage III, 16 stage Iva, and 25 stage IVb. An amount of 12 cases presented a papillary subtype, 85 colonic, 10 solid, and 32 mucinous subtypes. During follow-up, 60 patients developed local recurrence, 13 developed distant metastasis, and 54 died of the disease. The mean disease-free survival was 35 months (range 0–264); the mean overall survival was 55 months (range 0–460). Informed consent was obtained from the patients. All experimental protocols were approved by and carried out according to the Institutional Ethics Committee of the Hospital Universitario Central de Asturias and by the Regional CEIC from Principado de Asturias (approval number 2020.048).

Cell line ITAC-3 was established in 2011 from a previously untreated, colonic type T4N0 stage tumor [33] and was maintained in HuMEC serum-free culture medium (Gibco/Thermo Fisher Scientific Inc., Waltham, MA, USA). DNA and protein extraction and in vitro studies were carried out on passages 35–40.

### 4.2. Mutation Analysis

DNA from ITAC-3 cells and peripheral blood lymphocytes from the same patient were extracted with the Roche High Pure Template Preparation Kit (Roche Diagnostics GmbH, Mannheim, Germany). Whole exome sequencing (WES) and bioinformatic analysis were performed as described by Hieggelke et al. [24], and the mutation in gene *IRS4* exon 1 was confirmed with PCR using primers FW 5’-CCAATGGCTCCTCAAAATGT-3’ and RV 5’-AAGAGCCACCCTGAGGATTT-3’ run on a Simpliamp Thermal Cycler VXA24811 (Applied Biosystems/ Thermo Fisher Scientific, Inc., Waltham, MA, USA). The conditions were as follows: [95 °C for 5 min + (95 °C for 15 s, 58 °C for 1 min, 72 °C for 1 min) × 32 cycles + 72 °C for 7 min + 4 °C]. The PCR products were purified with Exo-BAP Mix (EURx Ltd., Gdansk, Poland) and sequenced with the ABI PRISM 3100 and 3730 Genetic Analyzers (Applied Biosystems/ Thermo Fisher Scientific, Inc., Waltham, MA, USA). Sense and antisense sequencing were performed for confirmation.

### 4.3. Immunohistochemistry

Three µm tissue sections from the TMAs and from a paraffin block containing cells from the ITAC-3 cell line were pre-treated in PT Link (Dako/Agilent Technologies Inc., Santa Clara, CA, USA) for 20–30 min at 95 °C, according to antibody and using epitope unmasking solution at pH 9 (EnVision FLEX Target Retrieval Solution High pH, Dako/Agilent Technologies Inc., Santa Clara, CA, USA). Then, samples were incubated with peroxidase (Dako/Agilent Technologies Inc., Santa Clara, CA, USA) for 5 min and subsequently 30 min with their corresponding antibody, then dissolved in Envision FLEX antibody diluent (Dako/Agilent Technologies Inc., Santa Clara, CA, USA).

The following antibodies were used: anti-p-AKT (Ser473, clone D9E; Dako/Agilent Technologies Inc., Santa Clara, CA, USA), anti-p-mTOR (Ser2448, clone 49F9; Cell Signaling, Danvers, MA, USA), anti-p-S6 (Ser235/236; Cell Signaling, Danvers, MA, USA), anti-p-4E-BP1 (Thr37/46, clone 236B4; Cell Signaling, Danvers, MA, USA), and anti-p-MAPK (Thr202/Tyr204; Cell Signaling, Danvers, MA, USA). Next, samples were incubated with EnVision FLEX/HRP (Dako/Agilent Technologies Inc., Santa Clara, CA, USA) for 30 min and stained with liquid DAB (Dako/Agilent Technologies Inc., Santa Clara, CA, USA) for 10 min. Finally, hematoxylin (Dako/Agilent Technologies Inc., Santa Clara, CA, USA) was used to stain cell nuclei for 8 min, and the samples were mounted with Entellan^®^ new for cover slipper (Millipore Corporation; Burlington, MA, USA).

Nuclear and/or cytoplasmic immunostaining was scored on a four-tiered scale for intensity (0 absent, 1 weak, 2 moderate, 3 strong) and percentage of positive tumor cells (1–25%, 26–50%, 51–75% and 76–100%), creating a final 3 level score by multiplying intensity and percentage scores: low (score 0–3), moderate (score 4–7), and high (score 8–12). In 50 of the 139 cases of ITAC, p-mTOR and p-ERK protein expression had been analyzed in a previous study [21]. The slides were evaluated in a double-blind manner by three observers, and discrepancies between the observers were resolved with a consensus review after simultaneous re-evaluation.

### 4.4. Cell Proliferation and Inhibitor Sensitivity Assay

Cell proliferation was evaluated using the RTCA iCELLigence System with E-Plates L8 (ACEA Biosciences, Inc., San Diego, CA, USA). This system uses impedance measurements from each individual well, reflecting the surface area grown by the tumor cells. Each well was seeded with 25,000 cells in 200 µL culture medium, and at 24 h, 350 µL culture medium with everolimus was added (Selleckchem, Madrid, Spain), selumetinib (Selleckchem, Madrid, Spain) was added, or a combination of both was added at 5 nM and 50 nM final concentration. The cells were monitored in real-time for 190 h after cell seeding. Cell proliferation was analyzed with RTCA Data Analysis Software version 1.0 (ACEA Biosciences, Inc., San Diego, CA, USA).

### 4.5. Western Blot

Protein extraction was carried out using a buffer consisting of 90% RIPA Lysis Buffer (Sigma-Aldrich; Darmstadt, Hesse, Germany), 1 mM sodium orthovanadate, 0.1 mM phenylmethylsulfonyl fluoride, phosphatase inhibitor PhosSTOP^TM^ 1 X (Roche, Basel, Switzerland), and Complete^TM^ Protease Inhibitor Cocktail 1 X (Roche, Basel, Switzerland). Then, samples were centrifuged at 13,000 rpm at 4 °C for 10 min, and the supernatant was collected and preserved at −20 °C until use. Protein quantification was analyzed with bicinchoninic acid technique (Pierce BCA Protein Assay kit; Pierce Biothecnology Inc., Waltham, MA, USA).

Electrophoresis of 30 µg total protein was performed on 4–15% Mini-PROTEAN^®^ Precast Gel (BioRad Laboratories Inc., Hercules, CA, USA) and then transferred to PVDF membranes (Millipore, Burlington, MA, USA), which were then blocked with 5% BSA in TBS-T (Tris-buffered saline with 5% BSA and 1% Tween-20). Membranes were incubated with the following antibodies during 24 h at 4 °C: anti-p-AKT (Ser473, clone D9E; Cell Signaling, Danvers, MA, USA), anti-AKT (clone C67E7; Cell Signaling, Danvers, MA, USA), anti-p-mTOR (Ser2448, clone D9C2; Cell Signaling, Danvers, MA, USA), anti-mTOR (clone 7C10; Cell Signaling, Danvers, MA, USA), anti-p-S6 (Ser235/236; Cell Signaling, Danvers, MA, USA), anti-S6 (clone 5G10; Cell Signaling, Danvers, MA, USA), anti-p-4E-BP1 (Thr37/46, clone 236B4; Cell Signaling, Danvers, MA, USA), anti-4E-BP1 (clone 53H11; Cell Signaling, Danvers, MA, USA), anti-p-MAPK (Thr202/Tyr204; Cell Signaling, Danvers, MA, USA), and anti-MAPK (Cell Signaling, Danvers, MA, USA). GAPDH (clone 6C5; Millipore, Burlington, MA, USA) was used as control to quantify and normalize protein levels. Next, membranes were incubated with corresponding secondary antibodies conjugated with horseradish peroxidase (Santa Cruz Biotechnologies, Dallas, TX, USA) during 1 h at RT. Finally, membranes were developed with Luminata^TM^ Forte (Millipore, Burlington, MA, USA) and Odyssey Fc Dual-Model Imaging System (LI-COR Biosciences, Lincoln, NE, USA). Images were acquired using the software Image Studio^TM^ Lite Quantification Version 5.2 (LICOR Bioscience, Lincoln, NE, USA). After analyzing the phosphorylated forms of the proteins, a stripping of the membranes was performed to study the total forms. To do this, the membranes were incubated with Restore^TM^ Western Blot Stripping Buffer (Thermo Fisher Scientific, Inc., Waltham, MA, USA) for 30 min at 37 °C.

### 4.6. Statistical Analysis

The Chi2 test was used to test possible associations between the immunohistochemical staining and clinico-pathological variables. Kaplan–Meier curves were plotted to assess survival using the Log rank test. Western blot quantification data are shown as the mean ± standard error of the mean, and comparison among groups was performed with one-way ANOVA with Bonferroni post hoc test. SPSS was used for statistical analysis IBM SPSS Statistics 25.0. (SPSS Inc., Chicago, IL, USA). Values of *p* < 0.05 were considered significant.

## Figures and Tables

**Table 1:** Clinical and follow-up data of 139 ITAC in relation to mTOR and ERK pathway protein expression.

		All	Tumor Stage					Histological Type				Recurrence		Metastasis		Patient Status		
			I	II	III	IVa	IVb	pap	col	sol	muc	No	Yes	No	Yes	Alive	DOD	DOC
All		139	134					139				134		134		139		
p-AKT	Low	32/67	5/17 (29%)	2/4 (50%)	14/28 (50%)	3/6 (50%)	8/12 (67%)	5/5 (100%)	16/44 (36%)	1/5 (20%)	10/13 (77%)	18/34 (53%)	14/33 (43%)	24/58 (41%)	8/9 (89%)	10/24 (42%)	17/33 (52%)	5/10 (50%)
	Medium	17/67	5/17 (29%)	1/4 (25%)	8/28 (29%)	1/6 (17%)	2/12 (17%)	0/5 (0%)	14/44 (32%)	1/5 (20%)	2/13 (15%)	8/34 (23%)	9/33 (27%)	16/58 (28%)	1/9 (11%)	6/24 (25%)	7/33 (21%)	4/10 (40%)
	High	18/67	7/17 (41%)	1/4 (25%)	6/28 (21%)	2/6 (33%)	2/12 (17%)	0/5 (0%)	14/44 (32%)	3/5 (60%)	1/13 (8%)	8/34 (24%)	10/33 (30%)	18/58 (31%)	0/9 (0%)	8/24 (33%)	9/33 (27%)	1/10 (10%)
	*p*		0.751					0.018 *				0.682		0.026 *		0.592		
p-mTOR	Low	45/139	6/31 (19%)	4/17 (24%)	14/45 (31%)	9/16 (56%)	8/25 (32%)	4/12 (33%)	28/85 (33%)	3/10 (30%)	10/32 (32%)	24/74 (33%)	17/60 (28%)	32/121 (26%)	9/13 (69%)	11/59 (19%)	21/54 (39%)	9/21 (43%)
	Medium	44/139	13/31 (42%)	6/17 (35%)	15/45 (33%)	2/16 (13%)	8/25 (32%)	3/12 (25%)	27/85 (32%)	3/10 (30%)	11/32 (34%)	20/74 (27%)	24/60 (40%)	40/121 (33%)	4/13 (31%)	21/59 (36%)	16/54 (30%)	7/21 (33%)
	High	50/139	12/31 (39%)	7/17 (41%)	16/45 (36%)	5/16 (31%)	9/25 (36%)	5/12 (42%)	30/85 (35%)	4/10 (40%)	11/32 (34%)	30/74 (40%)	19/60 (32%)	49/121 (41%)	0/13 (0%)	27/59 (45%)	17/54 (31%)	5/21 (24%)
	*p*		0.421					0.998				0.273		0.002 *		0.092		
p-S6	Low	29/118	5/25 (20%)	4/13 (31%)	8/39 (20%)	5/15 (33%)	6/23 (26%)	2/10 (20%)	17/74 (23%)	2/9 (22%)	8/25 (32%)	14/60 (23%)	14/55 (26%)	19/105 (18%)	9/10 (90%)	7/48 (15%)	16/51 (31%)	5/16 (31%)
	Medium	33/118	8/25 (32%)	3/13 (23%)	12/39 (31%)	5/15 (34%)	5/23 (22%)	2/10 (20%)	20/74 (27%)	3/9 (33%)	8/25 (32%)	18/60 (30%)	15/55 (27%)	32/105 (31%)	1/10 (10%)	12/48 (25%)	15/51 (29%)	6/16 (38%)
	High	56/118	12/25 (48%)	6/13 (46%)	19/39 (49%)	5/15 (33%)	12/23 (52%)	6/10 (60%)	37/74 (50%)	4/9 (45%)	9/25 (36%)	28/60 (47%)	26/55 (47%)	54/105 (51%)	0/10 (0%)	29/48 (60%)	20/51 (40%)	5/16 (31%)
	*p*		0.950					0.882				0.937		0.000 *		0.124		
p-4E-BP1	Low	31/118	4/25 (16%)	1/13 (8%)	11/39 (28%)	9/15 (60%)	5/23 (22%)	3/10 (30%)	17/74 (23%)	1/9 (11%)	10/25 (40%)	15/60 (25%)	15/55 (27%)	24/105 (23%)	6/10 (60%)	11/48 (23%)	15/51 (29%)	4/16 (25%)
	Medium	41/118	10/25 (40%)	6/13 (46%)	13/39 (33%)	2/15 (13%)	10/23 (43%)	6/10 (60%)	23/74 (31%)	5/9 (56%)	7/25 (28%)	23/60 (38%)	18/55 (33%)	39/105 (37%)	2/10 (20%)	20/48 (42%)	16/51 (31%)	5/16 (31%)
	High	46/118	11/25 (44%)	6/13 (46%)	15/39 (39%)	4/15 (27%)	8/23 (35%)	1/10 (10%)	34/74 (46%)	3/9 (33%)	8/25 (32%)	22/60 (37%)	22/55 (40%)	42/105 (40%)	2/10 (20%)	17/48 (35%)	20/51 (40%)	7/16 (44%)
	*p*		0.092					0.126				0.822		0.038 *		0.829		
p-ERK	Low	60/138	9/31 (29%)	5/17 (29%)	22/45 (49%)	10/16 (63%)	12/24 (50%)	4/12 (33%)	35/84 (42%)	4/10 (40%)	17/32 (53%)	27/73 (37%)	31/60 (52%)	48/121 (40%)	10/12 (84%)	21/59 (36%)	27/53 (51%)	10/21 (48%)
	Medium	39/138	12/31 (39%)	5/17 (29%)	13/45 (29%)	5/16 (31%)	3/24 (12%)	5/12 (42%)	24/84 (28%)	3/10 (30%)	7/32 (22%)	24/73 (33%)	14/60 (23%)	37/121 (30%)	1/12 (8%)	21/59 (36%)	11/53 (21%)	6/21 (29%)
	High	39/138	10/31 (32%)	7/17 (42%)	10/45 (22%)	1/16 (6%)	9/24 (38%)	3/12 (25%)	25/84 (30%)	3/10 (30%)	8/32 (25%)	22/73 (30%)	15/60 (25%)	36/121 (30%)	1/12 (8%)	17/59 (28%)	15/53 (28%)	5/21 (23%)
	*p*		0.121					0.864				0.224		0.015 *		0.420		

Legend. pap: papillary; col: colonic; sol: solid; muc: mucinous; DOD: died of disease; DOC: died of other causes; All statistical analyses by Pearson Chi2, *: significant.

## Data Availability

The datasets generated in the study are in the process of being deposited in a publicly available repository.

## Supplementary Materials

The following supporting information can be downloaded at: https://www.mdpi.com/article/10.3390/ijms242015110/s1.
