# Supplementary material for: Signaling Pathways mTOR and ERK as Therapeutic Targets in Sinonasal Intestinal-Type Adenocarcinoma
Source: Int J Mol Sci. 2023 Oct 12;24(20):15110. doi: 10.3390/ijms242015110 (PMC10606341; doi:10.3390/ijms242015110)
Supplement: Supplementary file 1 [file ijms-24-15110-s001.zip › ijms-2645019-supplementary.pdf]

Supplementary material

Signaling pathways mTOR and ERK as therapeutic targets in sinonasal intestinal-type adenocarcinoma.

Helena Codina-Martínez[1], Sara Lucila Lorenzo-Guerra[1], Virginia N. Cabal[1], Rocío García-Marín[1], Laura Suárez-Fernández[1], Blanca Vivanco[2], Paula Sánchez-Fernández[3], Fernando López[3], José Luis Llorente[3], Mario A. Hermsen[1]

Supplementary Figure S1. Wildtype (Wt) sequence and IRS4 frameshift deletion in exon 1 of IRS4 in cell line ITAC-3.

<u>**IRS4 exon 1 c.2244delC**</u>

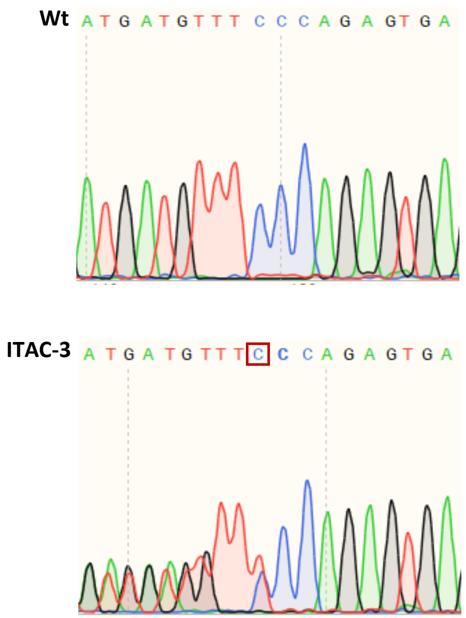

Supplementary Figure S2. Representative immunoblots for p-AKT, AKT, p-mTOR, mTOR, p-S6, S6, p-4E-BP1, 4E-BP1, p-ERK and ERK expression in cell line ITAC-3 after 48 hours with everolimus and selumetinib treatment (A). Expression ratios of phosphorylated and unphosphorylated AKT (B), mTOR (C), S6 (D). 4E-BP1 (E) and ERK (F) upon exposure to everolimus, selumetinib and combination of everolimus and selumetinib (all at 50 nM dosis), normalized to unexposed control cells. Experiments carried out in triplicate, expression ratios are shown as mean values ± standard error of the mean.

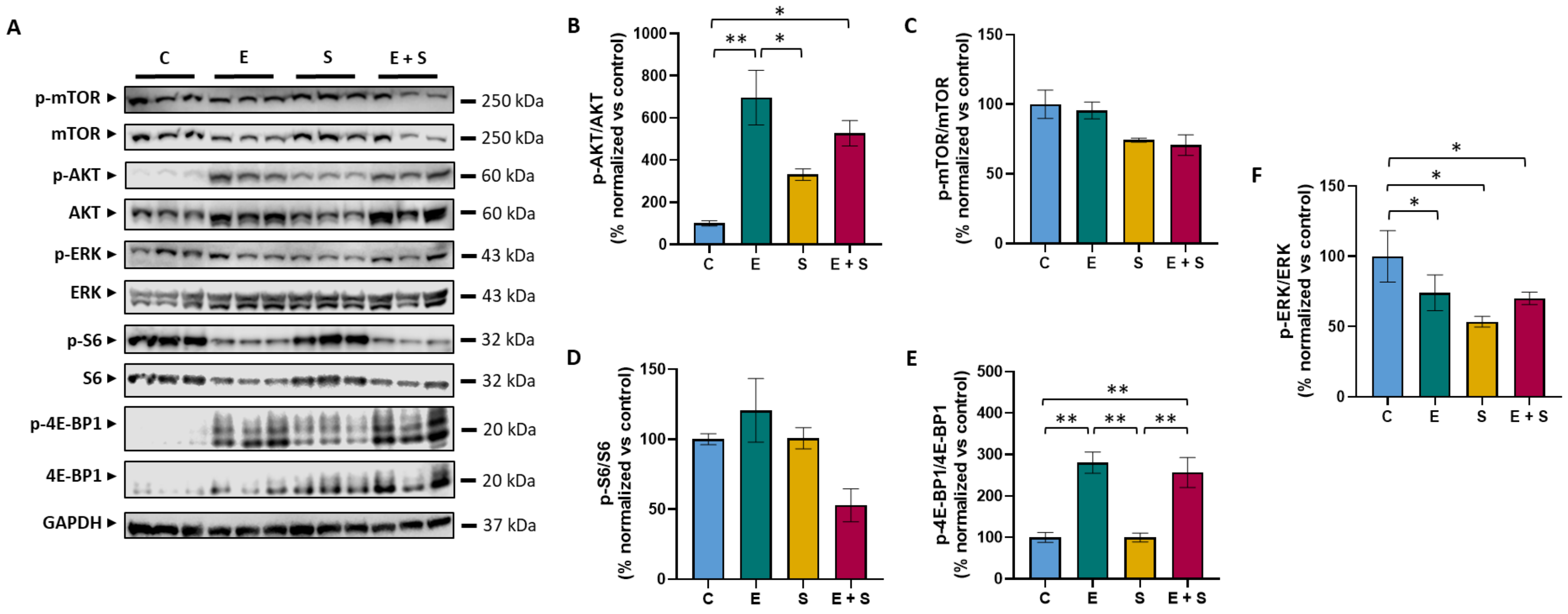

Legend. C: unexposed control cells; E: everolimus 50 nM; S: selumetinib 50 nM; E+S: everolimus 50 nM and selumetinib 50 nM; *: p=0.05-0.01; **: p=0.01-0.001; ***: p<0.001.
